# The cAMP-binding Popdc proteins have a redundant function in the heart

**DOI:** 10.1042/BST20130264

**Published:** 2014-03-20

**Authors:** Thomas Brand, Subreena L. Simrick, Kar Lai Poon, Roland F.R. Schindler

**Affiliations:** *Heart Science Centre, National Heart and Lung Institute, Imperial College, Hill End Road, Harefield UB9 6JH, U.K.

**Keywords:** cAMP, cardiac arrhythmia, caveolin 3, evolution, Popeye-domain-containing protein (Popdc), TREK-1, AS, antisense, AV, atrioventricular, Cav, caveolin, Epac, exchange protein directly activated by cAMP, PBC, phosphate-binding cassette, PKA, protein kinase A, Popdc, Popeye-domain-containing, SAN, sinoatrial node, SSS, sick sinus syndrome, t-tubule, transverse tubule

## Abstract

*Popdc* (Popeye-domain-containing) genes encode membrane-bound proteins and are abundantly present in cardiac myocytes and in skeletal muscle fibres. Functional analysis of *Popdc1* (*Bves*) and *Popdc2* in mice and of *popdc2* in zebrafish revealed an overlapping role for proper electrical conduction in the heart and maintaining structural integrity of skeletal muscle. Popdc proteins mediate cAMP signalling and modulate the biological activity of interacting proteins. The two-pore channel TREK-1 interacts with all three Popdc proteins. In *Xenopus* oocytes, the presence of Popdc proteins causes an enhanced membrane transport leading to an increase in TREK-1 current, which is blocked when cAMP levels are increased. Another important Popdc-interacting protein is caveolin 3, and the loss of *Popdc1* affects caveolar size. Thus a family of membrane-bound cAMP-binding proteins has been identified, which modulate the subcellular localization of effector proteins involved in organizing signalling complexes and assuring proper membrane physiology of cardiac myocytes.

## The Popeye-domain-containing gene family

**Figure 1:**
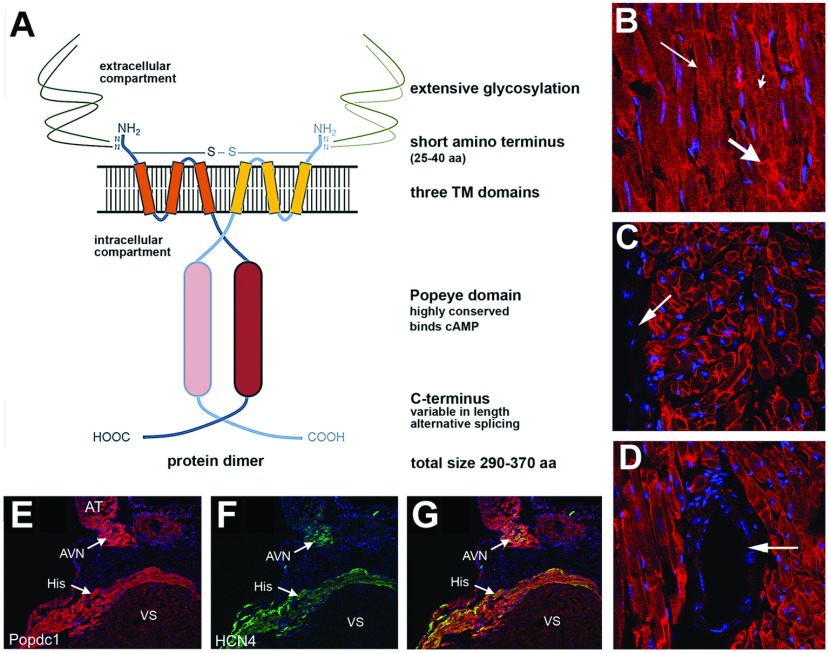
Model of Popdc proteins and expression of Popdc1 in the heart (**A**) Working model of Popdc proteins. The Popdc protein is found as a dimer in cells and consists of a short extracellular domain, which is subject to glycosylation, three transmembrane domains and a conserved Popeye domain in the intracellular part of the protein. The C-terminus is variable in length and subject to alternative splicing. aa, amino acids. (**B**–**E**) Immunohistochemical detection of Popdc1 in the adult mouse heart. Sections were counterstained with DAPI to label the nuclei. (**B**) Expression of Popdc1 in cardiac myocytes. Fat arrow, intercalated disc; thin arrow, lateral membrane; small arrow, t-tubules. (**C** and **D**) Lack of Popdc1 expression in the epicardium and subepicardium (arrow in **C**) and in coronary arteries (arrow in **D**). (**E**–**G**) The expression levels of Popdc1 in the atrioventricular node (AVN) and His bundle (His) are higher than in the ventricular septum (VS) and equal to the level in the atria (AT). Immunohistochemical staining of mouse hearts using antibodies directed against (**E**) Popdc1 and (**F**) HCN4 (hyperpolarization activated cyclic nucleotide-gated potassium channel 4). (**G**) Merge of Popdc1 and HCN4 staining.

The *Popdc* (Popeye-domain-containing) gene family has been discovered independently by two groups that aimed at identifying novel genes with cardiac-enriched expression pattern [1,2]. The *Popdc* gene family is strongly expressed in the heart and in skeletal muscle, and this expression pattern is observed for all vertebrate model organisms that have been studied to date. The gene family consists of three members, i.e. *Popdc1* (also known as *Bves*), *Popdc2* and *Popdc3* that display an overlapping expression pattern [3,4]. However, each member of the family displays distinct expression levels in different muscle tissues [5]. In humans, for example, *POPDC1* and *POPDC3* are expressed more abundantly in skeletal muscle, whereas *POPDC2* has a higher expression level in the heart [2]. The level of *Popdc1* expression in the mouse heart is higher in the atria and cardiac conduction system than in the ventricular working myocardium, whereas *Popdc2* is homogeneously expressed throughout the heart, but again with higher levels in the cardiac conduction system [6] (Figure 1). Apart from being expressed in striated muscle cells, *Popdc* genes are also expressed in smooth muscle tissue lining the gastrointestinal tract, bladder and uterus. *Popdc* genes are also present in some neurons of the central and peripheral nervous system, as well as in specific types of epithelial cells such as the pyloric epithelium of the stomach [7].

## Structure and biochemical properties of Popdc proteins

**Figure 2:**
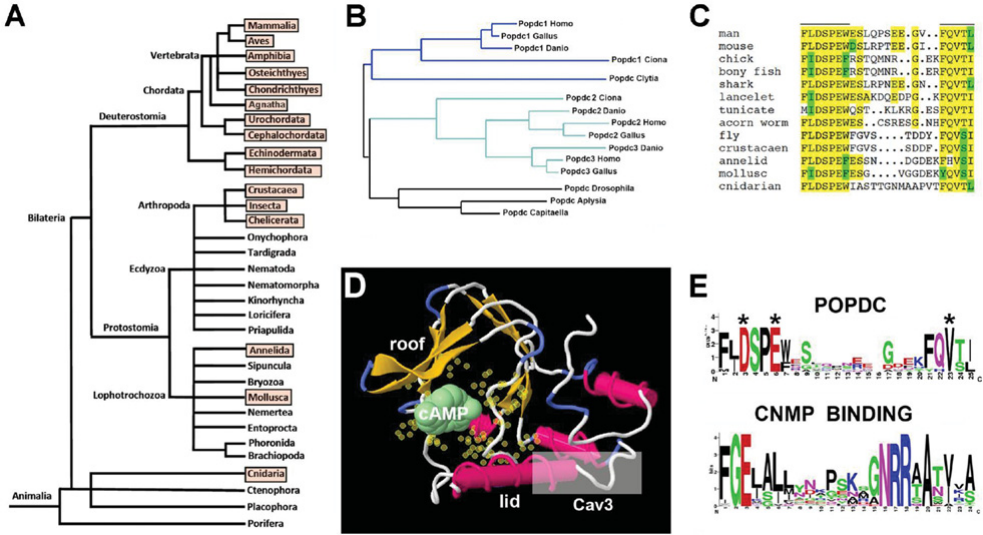
Evolution of the *Popdc* gene family (**A**) Animal phyla for which genomic sequences encoding Popdc proteins are present in the NCBI or Ensembl databases are boxed in red. (**B**) Phylogenetic dendrogram of Popdc protein sequences. Vertebrate Popdc proteins cluster in two groups: Popdc1 and Popdc2/3. Proteins found in basic chordates (*Ciona*) also distribute in these two subfamilies, whereas Popdc proteins of protostomes (*Drosophila*, *Aplysia* and *Capitaella*) form an independent subgroup equally distant from Popdc1 and Popdc2/3 subgroups. Significantly, however, the cnidarian (*Clytia*) Popdc protein appears to be orthologous to Popdc1, suggesting that Popdc1 represent the ancestral form of the protein family. (**C**) Protein sequence alignment of the PBC of Popdc proteins. Despite 650–850 million years of evolutionary distance between cnidarians and vertebrates, two sequence elements (FL/IDSPEW/F and FQVT/SL/I) are strongly conserved. (**D**) 3D model of the Popeye domain of human POPDC1. Similar to other cAMP-binding domains, the roof of the Popeye domain consists of a number β-strands, whereas the lid is α-helical and positions itself over the PBC in response to cyclic nucleotide binding and shields the ligand from solvent [36]. The residues of the PBC are depicted as yellow halos surrounding the cAMP molecule. The Cav3-binding site is also demarcated and located at the distal end of the lid domain. At this position, the binding site is likely to experience significant conformational changes in response to ligand binding. (**E**) Comparison of the consensus sequence of the PBC of Popdc proteins and the consensus sequence of cNMP-binding sites, which are aligned in PROSITE (http://prosite.expasy.org/PS00889). Note that the two consensus sequences do not resemble each other. In both cases, however, two stretches of conserved sequence motifs border a sequence of weak conservation. Amino acids labelled by a star in the Popdc sequence have been mutagenized and shown to be essential for cAMP binding [6].

Popdc proteins have been shown to be transmembrane proteins containing three hydrophobic helices. The proteins have a short extracellular N-terminus, which contains one or two N-glycosylation sites [8] (Figure 1). Glycosylation is quite extensive in these proteins and, because of this modification, Popdc1 with a predicted molecular mass of approximately 43 kDa runs on polyacrylamide gels with an apparent molecular mass of 58 kDa [2]. In the intracellular part of the proteins, the evolutionarily conserved Popeye domain (Pfam domain PF04831) is present, which consists of approximately 150 amino acids [2] (Figure 1). This domain functions as a cyclic nucleotide-binding domain [6]. Tertiary structure modelling revealed strong structural similarity to other cAMP-binding domains, whereas, at the sequence level, little homology is present and in particular the PBC (phosphate-binding cassette) is distinct (Figure 2). cAMP-affinity precipitation, radioligand-binding assay and FRET analysis demonstrate that Popdc proteins are able to bind cAMP, but not cGMP [6]. The binding affinity is approximately 10-fold higher than Epac1 (exchange protein directly activated by cAMP 1) and in the same range as that of PKA (protein kinase A). Two sequence motifs (FL/IDSPEW/F and FQVT/SL/I) are crucial for cAMP binding and are strongly conserved in evolution (Figure 2). Charge-to-alanine mutations of Asp^200^ in Popdc1 and the homologous residue (Asp^184^) in Popdc2 causes a massive reduction in cAMP binding [6]. Although the physiological relevance of the cAMP-binding ability of Popdc proteins has not yet been established, it has been shown that binding of cAMP to Popdc proteins has an effect on the interaction with the two-pore domain potassium channel TREK-1 [6] (see below).

At the C-terminus, the protein sequence is highly divergent among the family members, and different mRNA species are generated by alternative splicing. Popdc proteins form homodimers, which are stabilized by disulfide bonds [8,9]. Two conserved lysine residues at the C-terminal end of the Popeye domain mediate homodimerization [10]. Previous data, however, show that Popdc1 proteins lacking this sequence motif are still competent to dimerize, indicating the presence of other sequences elsewhere in the protein that also contribute [11].

## Genomic organization and evolution

Human *POPDC1* and *POPDC3* are localized in a tandem-organized manner on chromosome 6q21. This chromosomal organization is evolutionarily conserved and is found in all higher vertebrates, but is already present in primitive chordates [3,12,13]. Both genes display extensive co-expression [2], but also co-regulation in the diseased state [14–16]. Human *POPDC1* and *POPDC3* are separated by an intergenic region of approximately 20 kb. Interestingly, this locus appears to be transcribed from a bidirectional *POPDC1* promoter and, aside from the transcript encoding Popdc1, an antisense RNA (*POPDC1* AS) is also generated, which has sequence overlap in antisense orientation with *POPDC3*. An attractive hypothesis is that expression of *POPDC1* and *POPDC3* are controlled through a common control region and that the significantly lower expression levels of *POPDC3* are due to suppression by the *POPDC1* AS transcript.

In chicken, an mRNA was cloned, which contains sequences of both *POPDC1* and *POPDC3*, suggesting that gene boundaries are not strict [1,2]. This is possibly also occurring in a number of other species. Similar *Popdc3*–*Popdc1* fusion transcripts are predicted for a number of vertebrate species, suggesting a complex regulation of this genomic locus. The *Popdc2* gene is only present in higher vertebrates and is found on chromosome 3q13.33 in humans. *Popdc2* shows strong similarity to *Popdc3* at the protein sequence level, suggesting that these two genes have diverged only recently.

As mentioned above, three *Popdc* genes are found in vertebrates, whereas in lower chordates, two genes are present. In arthropods (insects, arachnids and crustaceans), *Popdc* genes are present; however, they do not cluster with vertebrate *Popdc* genes and form an independent subgroup (Figure 2). Although a number of protostome phyla have a single *Popdc* gene (molluscs and annelids), the majority of insects have up to three *Popdc* genes, with the exception of dipterans (*Drosophila*), which also have a single *Popdc* gene. Surprisingly, in crustaceans and arachnids, species are found with five and even eight genes. Thus gene duplications occurred several times during evolution of the *Popdc* gene family. Cnidarian species also have *Popdc* genes, suggesting that *Popdc* genes are already present in these primitive organisms at the base of metazoan evolution. Moreover, the cnidarian *Popdc* gene clusters with the vertebrate *Popdc1* group suggesting that *Popdc1* is the ancestral gene and that the paralogues *Popdc2* and *Popdc3* are subsequently generated by gene duplication during chordate (*Popdc3*) and early vertebrate (*Popdc2*) evolution.

## Loss-of-function mutations in mouse and zebrafish cause defects in striated muscle formation and function

All three members of the Popdc family are abundantly expressed in cardiac and skeletal muscle. In zebrafish, morpholino oligonucleotide-mediated knockdown of *popdc2* results in aberrant tail morphology with abnormal myotomal segment boundaries [17]. Furthermore, alignment of myofibrils is disturbed and ruptured myofibrils have been observed, which is probably due to aberrant formation of the myotendinous junctional complex [17]. In contrast with the developmental defects seen in zebrafish morphants, null mutants of *Popdc1* in the mouse develop normally [18,19]. However, a reduced regenerative capacity of muscles devoid of *Popdc1* has been observed. Therefore, in both mouse and zebrafish, depletion of *Popdc* genes causes a muscle defect. At present, however, the molecular basis for these phenotypes has not been established.

Depletion of *Popdc1* or *Popdc2* in the mouse and zebrafish model systems also results in severe cardiac arrhythmias [17]. In *Popdc1*^−/−^ as well as *Popdc2*^−/−^ mice, the presence of a stress-induced sinus bradycardia has been reported. At rest, null mutants do not differ in their heart rates from wild-type; however, when subjected to either physical or mental stress, the mean heart rate of the null mutants is significantly lower. This stress-induced bradycardia is caused by an increase in the number of sinus pauses [temporary failure of SAN (sinoatrial node) activity] occurring during and after physical exercise, resulting in massively increased heart rate variability and a significant reduction of the mean heart rate [6]. In both mouse models, the bradycardia develops in an age-dependent manner.

In addition to the electrophysiological phenotype, structural degeneration of the primary pacemaker, the SAN has been described in older *Popdc1*^−/−^ and *Popdc2*^−/−^ mice [6]. Interestingly, this loss of tissue is more prominent in the inferior part of the SAN, which is particularly important for pacemaking after adrenergic stimulation [20]. Furthermore, single SAN cells of *Popdc1*- and *Popdc2*-null mutants display a reduced number of cellular extensions and therefore the SAN tissue has a more compact appearance. There is also evidence for a loss of pacemaker cells since the volume of the sinus node appears to be reduced and the number of fibroblast and extracellular matrix has increased [6].

These findings are reminiscent of the SSS (sick sinus syndrome) in humans, which affects mainly the elderly. Similar to the observed phenotypes in *Popdc*-null mutants, hearts of SSS patients have difficulties adapting their beating frequency to the physiological demands [21]. It has therefore been hypothesized that, in some SSS patients, the disease may be caused by abnormal *Popdc* gene expression or function. However, at present, little is known about the role of *POPDC* genes in human heart disease. It has recently been reported that expression of *POPDC1* and *POPDC3*, and, to a lesser extent, also *POPDC2*, are reduced in failing hearts compared with normal control hearts, but the degree of down-regulation varies between patients [14].

In zebrafish, morpholino oligonucleotide-mediated knockdown of *popdc2* results in misshaped heart chambers with reduced myofibrillar content, lack of trabeculation and embryonic heart failure characterized by large pericardial oedema and defective heart looping [17]. When lower morpholino concentrations are used, the morphological abnormalities are reduced; however, a bradyarrhythmic phenotype with cardiac conduction abnormalities and a 3:1 or 2:1 AV (atrioventricular) block is observed. With the help of SPIM (single-plane illumination microscopy), Ca^2+^ transients were analysed in different compartments of the heart, revealing that the morphants show strong variability in action potential duration in both atrium and ventricle [17]. Similar to null mutants in mice, some embryos display a SAN block and a dramatic lowering of the heart rate. At present, it is not clear whether the observed defects are due to a role for *popdc2* in the development of cardiac conduction tissue or to a direct function of the gene in action potential generation.

## Popdc proteins interact with TREK-1 and Cav3

Relevant for the observed arrhythmia phenotypes in zebrafish and mouse mutants is the recent finding that Popdc proteins interact functionally with TREK-1 [6]. TREK-1 is a member of the two-pore domain potassium channel (K_2_P) family [22]. The open probability of this ion channel is mainly regulated via its intracellularly localized C-terminus by a number of different physiological stimuli (reviewed in [23]). Expression levels of TREK-1 in the atria are higher than in the ventricles [24]. By immunostaining of ventricular cardiac myocytes, TREK-1 has been observed in longitudinal stripes at the cell surface [25–28]. It is believed that the main function of TREK-1 in the heart is to act as a stretch sensor. Furthermore, a role in modulating ANP (atrial natriuretic peptide) secretion has been suggested [24]. The *Trek-1*-null mutants in mice have a normal lifespan and no obvious morphological changes of the heart were reported [29].

Using *Xenopus* oocytes as a heterologous expression system, TREK-1 has been identified as a specific interaction partner of Popdc1 [6]. Co-expression of both Popdc1 and TREK-1 results in a current, which is approximately 2-fold higher than in the absence of Popdc1. Similarly, the TREK-1 current is also increased in the presence of Popdc2 or Popdc3. This interaction is specific for TREK-1, as conductivity of the closely related TASK-1 channel is not enhanced. The increase in TREK-1 current is accompanied by an approximately 2-fold higher membrane localization of TREK-1. It is therefore believed that Popdc proteins modulate TREK-1 trafficking. Importantly, in the presence of theophylline, which increases cAMP levels, the effect of Popdc co-expression is abolished and no increase in TREK-1 conductivity is observed [6]. On the basis of this molecular interaction, a bimolecular FRET sensor using Popdc1–CFP and YFP–TREK-1 was designed. Increasing cAMP levels causes an immediate change in the FRET ratio, being independent of PKA activity [6]. However, at present, the effect of this instantaneous conformational change of the Popdc–TREK-1 complex on channel properties is not understood.

Lipid rafts are cholesterol- and sphingolipid-rich subcompartments of the cell membrane. Specialized forms of lipid rafts are caveolae (Latin for ‘little caves’), which are flask-like invaginations of the cell membrane [30]. Important protein components of caveolae are cavins and caveolins. Caveolins are approximately 20 kDa in size, are able to form oligomeric complexes and insert into the plasma membrane asymmetrically, thereby contributing to the flask-like shape of caveolae [31]. Three caveolin isoforms exist (Cav1, Cav2 and Cav3). Cav3 is the muscle-specific isoform, which localizes to the sarcolemma in skeletal muscle fibres and in sarcolemma and t-tubules (transverse tubules) in cardiac myocytes [32]. Caveolae play various physiological roles, e.g. in vesicular trafficking, mechanosensation and transduction, and in signalling processes such as β-adrenergic signalling, and therefore in the control of cAMP production [33]. Mutations in the *CAV3* gene are associated with neuromuscular diseases such as limb-girdle muscular dystrophy and rippling muscle disease, and with congenital LQT (long QT) and sudden infant death syndromes [34].

Cav3 has recently been identified as an interaction partner of Popdc1 [35]. In the absence of *Popdc1*, caveolae in cardiac myocytes were altered in number and size. These might contribute to the observed ischaemia/reperfusion vulnerability of *Popdc1*-null mutant hearts, but may also be involved in causing the cardiac arrhythmia phenotypes in *Popdc1* mutants [35]. Popdc1 was co-sedimented with Cav3 from membrane fractions, and the binding site for Cav3 is localized to the lid region of the Popeye domain (Figure 2).

## Two working models of Popdc protein function in striated muscle

**Figure 3:**
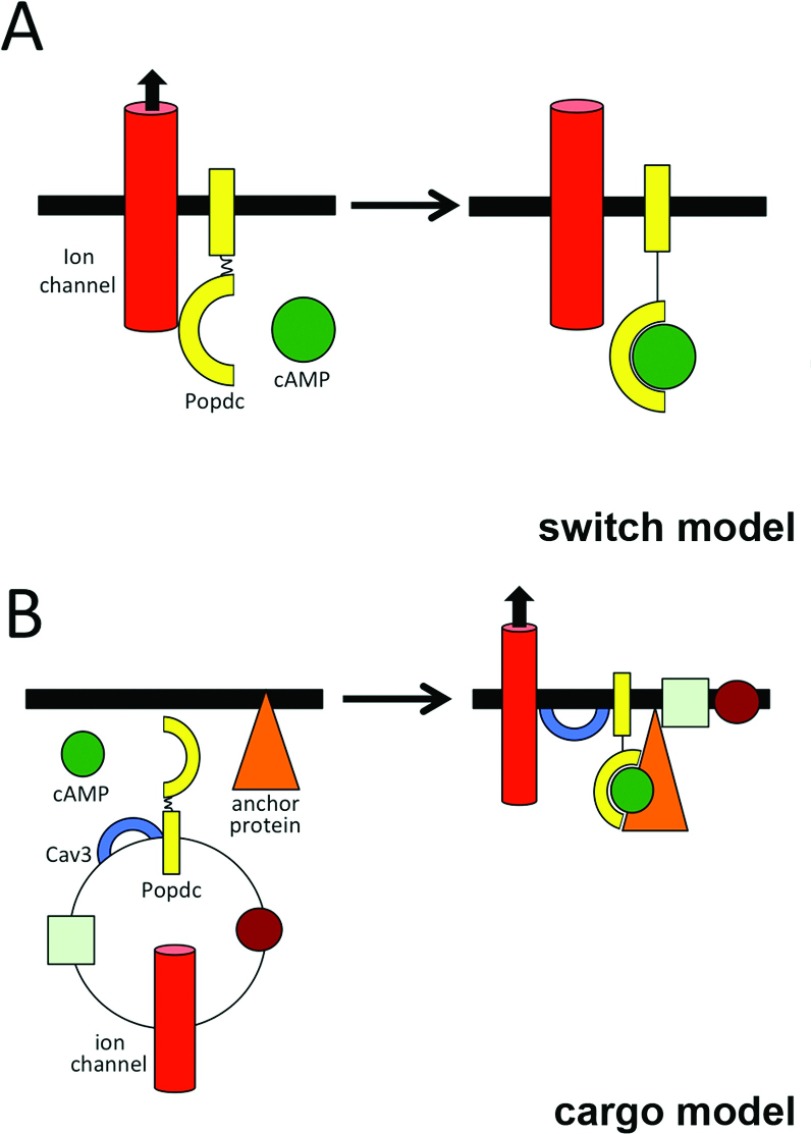
Models of Popdc protein function (**A**) Switch model: Popdc protein might act as a switch that activates or inactivates proteins with which it forms a complex. The example depicted is the potassium channel TREK-1, which forms a complex with Popdc protein. Binding of cAMP may induce a change in protein conformation, leading to a modulation of the open probability of the associated ion channel. (**B**) Cargo model: Popdc proteins are found in cytoplasmic vesicles and may regulate the transport of effector proteins to the plasma membrane, which may also involve Cav3, with which Popdc proteins form a complex. Both models are supported by protein–protein interaction data of Popdc proteins with TREK-1 and Cav3 [6,35].

Two working models of how Popdc proteins might act in muscle cells could be envisaged (Figure 3). The ‘switch’ model suggests that ligand binding to the Popdc proteins will cause conformational changes such as movement of the lid domain over the PBC as has been proposed for other cAMP-binding proteins [36]. Given, for example, that the Cav3-binding site is localized to the lid region makes it likely that ligand binding will have an immediate impact on the Cav3–Popdc1 interaction. Indeed, our FRET analysis of Popdc1 and TREK-1 interaction support the concept of a rapid ligand-mediated modulation of protein–protein interactions [6]. In this regard, cAMP binding triggers a switch that activates or inactivates the interacting protein. Such a function might enable the cell to respond rapidly to changes in cAMP levels.

An alternative view is represented by the ‘cargo’ model (Figure 3), which suggests that one of the major functions of Popdc proteins is to facilitate membrane transport. This concept finds support from our analysis of the interaction of TREK-1 and Popdc1 in *Xenopus* oocytes. Co-expression of both proteins enhances the presence of TREK-1 at the plasma membrane. Moreover, Popdc proteins are not localized exclusively at the plasma membrane, but also in an intracellular vesicle compartment [9]. It therefore could be envisaged that membrane proteins such as ion channels or membrane-associated proteins are recruited to the plasma membrane in response to an increase in cAMP, which causes a ligand-induced change in protein conformation, making it possibly competent to bind to anchor proteins. Both models are supported by experimental data, therefore we think that they are not mutually exclusive, but may represent different aspects of the complex roles that Popdc proteins might play in cardiac and skeletal muscle cells.

## Outlook

Popdc proteins are an evolutionarily conserved class of proteins with an abundant presence in the plasma membrane of skeletal muscle and heart. However, their specific roles in muscle membrane physiology is at present only poorly understood. It is likely that, in addition to Cav3 and TREK-1, other membrane and membrane-associated proteins are interacting with Popdc proteins. The bradyarrhythmia phenotype that has been observed in *Popdc*-null mutants has also been observed in the case of the sodium channel SCN5A and the anchor protein ankyrin B [37,38]. Thus these proteins are strong candidates as interaction partners of Popdc proteins. A number of proteins, including ZO-1 (zonula occludens 1) [39], guanine-nucleotide-exchange factor GEFT [40], Vamp2/3 (vesicle-associated membrane protein 2/3) [41] as well as Ndrg4 (N-Myc downstream regulated gene 4) [42], have been identified as novel interaction partners for Popdc1. However, these interaction partners have been mostly characterized in epithelial cells, and their importance in skeletal and cardiac muscle cells is currently uncertain.

It also needs to be stressed that the identification of an additional mediator of cAMP signalling in vertebrates has some important implications. It is likely that Popdc proteins might actually mediate some effects of cAMP which are thought to involve Epac or PKA. In order to clarify this point, it is important to re-evaluate the specificity of agonists and antagonist currently thought to specifically activate or inhibit Epac or PKA and, if possible, to identify ligands that specifically activate Popdc proteins.

Another important avenue of research in the future is to answer the question of whether cardiac and skeletal muscle pathologies that are associated with *Popdc*-null mutations in animal models are also found in patients. In GWAS (genome-wide association studies), *Popdc1* has so far not been associated with any major cardiac disease. However, given the complexity of the *Popdc* gene family characterized by an overlapping expression pattern and functional redundancy, it might be difficult to find associations with inherited forms of cardiac and skeletal muscle disease. Thus dedicated screens to identify *Popdc* gene mutations in patients with striated muscle dysfunction are more likely to be able to define the human correlate of the mutant phenotypes in animal models.
